# Research Ethics in Training Curricula of Cameroon Health Training Institutions: A Cross-Sectional Study

**DOI:** 10.7759/cureus.59567

**Published:** 2024-05-03

**Authors:** Ketina Hirma Tchio-Nighie, Cavin Epie Bekolo, Frank Forex Kiadjieu Dieumo, Charles Kouanfack, Jerome Ateudjieu

**Affiliations:** 1 Department of Health Research, Meilleur Accès Aux Soins de Sante (M.A. SANTE), Yaoundé, CMR; 2 Department of Public Health, University of Dschang, Dschang, CMR; 3 Department of Infectious Disease, Central Hospital of Yaoundé, Yaoundé, CMR; 4 Division of Health Operations Research, Cameroon Ministry of Public Health, Yaoundé, CMR

**Keywords:** medical education, curricula, health training institutions, cameroon, research ethics

## Abstract

Introduction

The quality of protection for research participants in Africa is still marked by the lack of trained actors in this area. The study was conducted to assess the availability of health research ethics in the curricula of health-related sciences training institutions in Cameroon.

Methods

The study involved a cross-sectional analysis to describe the training curricula on research ethics in health training institutions in Cameroon. Data were collected using a structured questionnaire that was administered face to face to the heads of institutions in late 2020.

Results

Twenty-one health training institutions were identified, and 18 (85.71%) participated. Health research ethics courses were present in the curricula of 16 (88.88%) of the institutions. Lectures were either a standalone module or part of a module in 14 (77.78%) institutions. The three first topics covered in the courses were the fundamental principles of ethics, the role of the ethics committee in the protection of research participants, and respect for research participants. A total of 14 (77.78%) institutions declared ethical clearance mandatory before the implementation of students’ thesis protocols. Eight (50.00%) training institutions declared having at least one qualified lecturer to deliver training in research ethics evaluation. The organization of the training of lecturers in delivering lectures on research ethics was declared to be the main assistance needed.

Conclusions

The delivery of research ethics education in Cameroon’s healthcare institutions is still limited by the fact that it does not cover all eligible populations, is not standardized, and does not yet promote the practice of requiring all student protocols to undergo preethical review prior to implementation. These points should be taken into account by the authorities in charge.

## Introduction

Research regarding human health is essential in identifying and responding to human-related health problems in general and represents a vital driver of development. Its implementation has become increasingly prominent in finding ways to design, conduct, expand, and sustain effective and equitable health policies, programs, and related interventions [1,2]. Health research is implemented by a variety of actors in different fields to contribute to similar objectives.

Research has shown its beneficial contribution to improving people’s health [3]. However, its planning and implementation can expose participants to risks that can compromise their autonomy, confidentiality, and family and social dignity, as well as risks of physical and economic harm at several levels of severity [4]. Similarly, because of the interests and stakes involved, the implementation of a research project can expose research teams to risks of misbehavior, exploitation, and injustice that can compromise the results or lead to frustration for those involved [4]. It is in response to these risks to research participants and team members that international and national guidelines and regulations have been put in place [5-7]. The application of these guidelines involves a number of actors, including members of ethics committees, researchers, managers of research and health institutes hosting research projects, sponsors, regulatory authorities, and research participants [6,8-10]. During their training and career development, students who are to work in the health and health research sectors will not only conduct research as part of their training but will also be involved throughout their career in playing a more or less important role in developing or updating regulations for the protection of research participants or in applying these regulations to improve standards of protection for research participants [11]. These expected missions have supported the recommendation of including in the training curricula of medical students and those trained to work in the health or research sectors training on health research ethics [12].

To date, there are no international guidelines on the minimum initial training expected for each training cycle for different healthcare professionals. As a result, the curriculum for basic training in research ethics for healthcare professionals and the objectives and content of this training may vary from one training institution to another and from one training cycle to another [13,14]. This may explain why health personnel training programs in research participants’ protection have been documented to vary in different African universities and, in some circumstances, to be limited in coverage and content [3,13-15].

This situation is not likely to prepare healthcare professionals to play their role in protecting research participants during their training and in their professional lives, nor is it conducive to improving ethical standards for research conducted in universities and research institutions. There is a need to make available the evidence that can guide universities’ decision-makers in improving this situation, but to the best of our knowledge, the current state of the literature reveals a paucity of such evidence, both in Cameroon and in many other African countries.

The present study is proposed to contribute to generating a minimum of information on the situation of training curricula in health training institutions for different health professionals in Cameroon and to generate data that can guide the planning of actions to improve the coverage and response to the training needs of health personnel regarding the protection of research participants and actors.

## Materials and methods

Study design

A cross-sectional study targeting health training institutions across Cameroon was carried out from November to December 2020. Data were collected by trained surveyors using a structured questionnaire administered face to face to the heads of these institutions. Data on their training content and human resources regarding research ethics were collected.

Study setting and period

The study was carried out in private and public health training institutions in Cameroon that were listed on the websites of the Cameroon Ministries of Higher Education and Public Health. Data were collected from November to December 2020.

Study population

Any institution providing training in medicine and/or biomedical sciences and officially recognized by the Ministry of Higher Education and the Ministry of Health of Cameroon was eligible for this study. These were either private or public institutions. All nonfunctioning health training institutions and those who did not consent to participate were excluded from the study.

Sampling

Exhaustive sampling was used. All health training institutions offering training in medicine and/or biomedical sciences and officially recognized by the Ministry of Higher Education and the Ministry of Health of Cameroon were targeted.

Data collection

The study questionnaire was developed based on the study objectives and consisted of predefined questions with predefined response options. The questionnaire was integrated into the Kobo Toolbox platform and deployed on smartphones for data collection. The questionnaire was pretested with three lecturers before the study was implemented. The main variables collected included the availability of research ethics in the institution’s training program, the contents, and the availability of lecturers.

Data management and analysis

Data were collected from the heads of institutions using a structured questionnaire administered by supervised surveyors. Data collected were checked at two levels: firstly by the field-by-field supervisors before transmission to the cloud server, and secondly at a second level by the study data management team. At the second level, the consistency and completeness of the data were checked and corrected. A final cleaning and cross-checking were done prior to analysis. Data analysis was done using Epi Info 7.2. The analysis done was purely descriptive, with an estimation of the proportion of private and government health training institutions, the proportion of health training institutions offering training courses in health research ethics, the proportion of health training institutions having lecturers in health research ethics, and the proportion of health training institutions expressing particular needs.

Ethical considerations

The study protocol was approved by the Cameroon National Ethics Committee for Human Health Research (N°2020/10/1305/CE/CNERSH/SP). All participants were informed of the study, and their consent was obtained prior to the administration of the questionnaire. A security and restricted access system was set up for data protection.

## Results

Characteristics of respondents

Out of 22 health training institutions targeted, 21 were reached, of which 18 (85.71%) accepted to participate in the study. Ten (55.56%) were private institutions, and eight (44.44%) were public institutions. The main areas of training in these institutions included biomedical sciences, medicine, and public health (Table 1).

**Table 1:** Characteristics of health training institutions

Characteristics	Frequency (n)	Proportion (%)
Type of institution		
Private	10	55.56
Public	8	44.44
Total	18	100
Health training areas		
Medicine	4	22.22
Pharmacy	1	5.56
Biomedical sciences	6	33.33
Nursing	3	16.67
Medical/health profession	2	11.11
Public health	4	22.22
Biology	2	11.11
Physiotherapy	2	11.11
Basic sciences	2	11.11
Other	1	5.56

Availability of research ethics courses

Health research ethics courses were present in the curricula of 16 (88.88%) of the targeted health institutions. Lectures were either a standalone module or part of a module in 14 (77.78%) institutions, while they were offered as a topic in some courses in two (11.11%) institutions. Table 2 presents the distribution of the availability of health research ethics in training institutions. Most schools (61.11%), especially public ones, introduced ethics courses at the master level. Table 2 presents the characteristics of health research ethics courses offered in health training institutions.

**Table 2:** Availability of health research ethics courses in health training institutions * Some schools teach health research ethics at more than one level.

	Total		Private		Public	
	Frequency (n)	Proportion (%), N = 18	Frequency (n)	Proportion (%), N = 10	Frequency (n)	Proportion (%), N = 8
Availability of health research ethics lectures						
As a module or part of a module	14	77.78	9	90	5	62.5
As a topic in a course	2	11.11	1	10	1	12.5
None	2	11.11	0	-	2	25
The level at which health research ethics is introduced*						
Bachelor	10	55.56	7	70	3	37.5
Master	11	61.11	6	60	5	62.5
Doctorate	6	33.33	3	30	3	37.5
Training objectives						
Empowering students to protect research participants	12	66.67	8	80	4	50
Empowering students in research ethics review	9	50	7	70	2	25
Familiarize students with research ethics	12	66.67	7	70	5	62.5
Other	2	11.11	2	20	0	0
Availability of lecturers (n = 16)						
Trained in research ethics	8	50	4	40	4	50
Offering courses based on experience	8	50	7	70	1	12.5

Contents of health research ethics courses offered in health training institutions

Table 3 presents the contents of health research ethics courses offered in health training institutions. The main topics covered in these schools were “Fundamental principles of ethics,” “Role of the ethics committee in the protection of research participants,” and “Respect for confidentiality,” while the least covered were “Misconduct in research (plagiarism, ghost author, safari author, and data manipulation),” “Role of the sponsor in protecting research participants,” and “Social value.”

**Table 3:** Contents of health research ethics courses offered in health training institutions

Content of the modules	Frequency (n)	Proportion (%), N = 18
			Fundamental principles of ethics	12	66.67
Role of the ethics committee in the protection of research participants	10	55.56
Respect for research participants	9	50
Respect for confidentiality	9	50
Equity in the selection of research participants	8	44.44
Regulation of research involving humans	8	44.44
The informed consent process	8	44.44
Assessment of the benefit and risk	7	38.89
Research ethics review system in Cameroon	7	38.89
Role of the researcher in protecting research participants	7	38.89
The scientific validity of a research project	6	33.33
Ethical issues in qualitative research	6	33.33
Participant autonomy	6	33.33
The social value of research	6	33.33
Role of the sponsor in protecting research participants	5	27.78
Misconduct in research (plagiarism, ghost author, safari author, and data manipulation)	4	22.22

Ethical clearance and thesis protocols’ implementation

**Figure 1:**
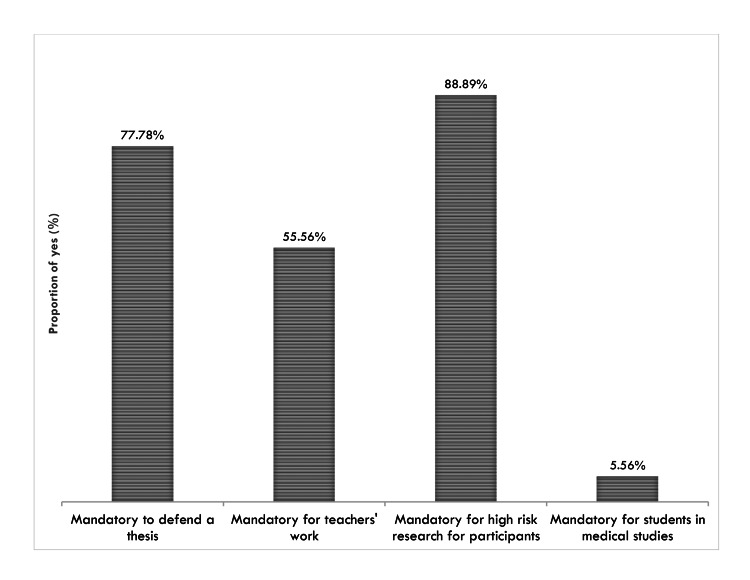
Conditions under which ethical clearance is mandatory in Cameroon health training institutions

Fourteen (77.78%) of the surveyed institutions declared ethical clearance mandatory before the implementation of students’ thesis protocols. Figure 1 presents the levels at which ethical clearance was declared to be mandatory in health training institutions.

Assistance needs to improve the provision of health research ethics in health training institutions

**Figure 2:**
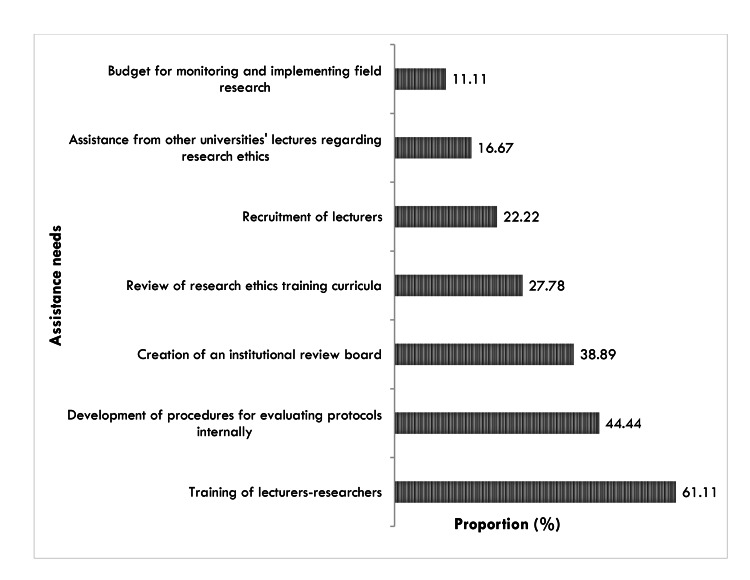
Major assistance needs of Cameroon health training institutions regarding health research ethics provision

The institutions reported that the major areas for which they needed support were the following: the training of lecturers (61.11%), the development of procedures for evaluating research protocols internally, and the creation of institutional review boards (Figure 2).

## Discussion

The study was conducted to explore the availability and content of health research ethics curricula in health training institutions in Cameroon.

Health research is a field that is implemented with the aim of developing new knowledge and contributing to the amelioration of human lives. Its implementation is framed by respect for ethical considerations to ensure its proper application [15]. The majority of people involved in human health research are graduates of training courses in the health sector. To this end, it is expected that during their training, where most conduct a thesis involving human participants, notions of ethics related to such research are instilled in them. The present study assessed the availability of courses on health research ethics in health training institutions in Cameroon. Results reveal that 88.88% of surveyed health training institutions offer training in health research ethics. Despite the place of health training institutions in the upbringing of researchers, very few published studies assessed this indicator, even though some studies documented the effect of training on the improvement of ethical practices [11,14,16]. The fact that not all institutions offer this training is a cause for concern, as it means that current and future healthcare staff trained in these institutions do not receive the knowledge they need to ensure the protection of research participants during the research projects they implement or are involved in. Even though some institutions that do not offer this training can catch up during interactions between students and thesis supervisors, this situation can lead to the observed conduct of unethical study and practices, including the implementation of research projects without an application for ethical approval and failure to respect informed consent and confidentiality processes. The introduction of guidelines making it compulsory to include research ethics courses in the training curriculum for medicine students should help improve this situation.

Meanwhile, it is documented and seems important to administer health research ethics courses to students in health training institutions, but the question of whether it is appropriate to offer these courses remains. We investigated in the present study the levels at which courses were offered. It was revealed from the present study that most of the training courses in research ethics are delivered at the master’s and doctorate levels, with few at the bachelor’s level. This diversity of course delivery levels is explained by the fact that there are no up-to-date academic guidelines indicating at what level these courses should be delivered. Similarly, to the best of our knowledge, there are no studies to indicate at what level these courses should be delivered to achieve the best return on participant protection learning. The study did not collect data on the reasons for the level at which the course is delivered, but we can deduce that the choice of delivering the majority of these courses at the master’s or doctoral level is explained by the fact that this course is included in the research courses, which are given more at these levels. If the argument of the importance of conducting research at the undergraduate level is taken on board by most training institutions, we should expect to see more of these courses being delivered at this level of education [17]. We believe that since one of the major objectives of this course is to prepare students for the protection of research participants during the planning and implementation of their theses, the timing of this course should be adapted to ensure that it is given before and within a timeframe that allows it to best contribute to this objective.

Submitting the application for ethical clearance and the interaction for the ethical review process is a real-life application of the training received in research ethics and, at the same time, an indicator of the training institute’s commitment to the protection of research participants. The fact that this is only compulsory in 83.3% of training institutions highlights the need for action with these institutions. It is well known that the submission of ethical clearance requests should not be an exception for student theses, as they correspond to the definition of human health research adopted by national and international regulations [18]. The observed situation in the actual study is not new and reflects that of developed countries, marked by a gradual increase in research education as the importance of teaching research ethics in health training schools evolves [19]. This highlights the need to set up an advocacy program to accelerate the awareness of those in charge of health school training programs.

The objectives and content of teaching research ethics should be adapted to the vision. The present study reveals a diversity of objectives and content, characterizing the nonstandardization of the teaching to be offered in this area. A review of studies carried out in various contexts reveals a similar situation throughout the world [20]. This can be explained either by the diversity of teaching objectives or by the diversity of implementation contexts. In any case, given that ethical principles depend little on national contexts, requiring students to be prepared to implement them in different contexts, it is advisable to recommend a minimum standard of objectives and content to be found in all health school training curricula [21].

Health research ethics represents a field in its complexity with various components. Thus, the administration of the course needs to be done by trained and experienced actors. The single most important problem raised for the provision of health research ethics courses in institutions was the lack of qualified lecturers in health research ethics. The problem is critical in half (50.0%) of the schools offering health research ethics, where there are no qualified health research ethics lecturers at all. This situation is similar to findings from Zambia, where up to 31% of respondents (lecturers) reported having never received any training in ethics, and in a study conducted in Turkey, there were no bioethics or medical ethics professors in 64.8% of medical schools [11,12]. This situation needs to be addressed urgently, particularly in private institutions where the problem is more critical. The absence of trained lecturers may not directly imply the poor quality of lectures offered in these health training institutions, as half of them declare to have lecturers who administer the courses based on experience. Even though experience may be a contributing factor to the quality of the courses, the standardization and basics may not necessarily be maintained, thus raising the need for training lecturers in charge of the courses. Along the same line, the need for trained lecturers was declared the most frequent need identified by health training institutions to improve the provision of health research ethics in their institutions and thus should really be taken into account when addressing the identified gaps.

The present study presents some limitations. The sampling method led to the exclusion of some health training institutions that were not officially registered in the appropriate registries. Nevertheless, we do not believe that their exclusion or inclusion would significantly change the direction of interpretation of the results. Also, not all eligible institutions were reached or consented to participate. We cannot predict what would have been the effect of their response on the proportion of institutions exposed to health research ethics training.

## Conclusions

This study highlights important gaps in the provision of health research ethics in health training institutions in Cameroon that need to be addressed. It reveals a diversity and incomplete coverage of schools by this course, a lack of standardization in terms of level, objectives, and teaching content. It also brings to light the fact that in some health institutions, the submission of students’ thesis protocols for ethical evaluation and approval before implementation is not mandatory. The main need identified by the included training institutions was the training of lecturers in charge of delivering research ethics. To improve the situation, there is a need to develop and implement guidelines for the basic training of future research and healthcare staff. These guidelines should cover the definition of the categories of students who should benefit from training in research ethics, the minimum qualifications of the lecturers who should deliver such training, the vision, objectives, and academic level of such training, and the obligation to submit all students’ research protocols involving human beings to ethical appraisal and approval prior to implementation. Future research should document the application of ethical principles when implementing student protocols.
